# Principles for Responsible AI in Health Professions Education, Research, and Care: Health CARE-AI (Contextual, Accountable, Responsible, and Equitable Artificial Intelligence) Framework Delphi Consensus Study

**DOI:** 10.2196/91626

**Published:** 2026-07-22

**Authors:** Lyn K Sonnenberg, David Wiljer, Muhammad Mamdani, Victor Do, Brandon Tang, Babar Haroon, Bizav Jaffer, Jerry Maniate

**Affiliations:** 1 Equity in Health Systems (EqHS) Lab, Bruyère Health Research Institute, 43 Bruyère Street, Ottawa, ON, K1N 5C7, Canada, 1 613-562-6262; 2 Department of Pediatrics, Faculty of Medicine and Dentistry, University of Alberta, Edmonton, AB, Canada; 3 Education Technology Innovation, Digital, University Health Network, Toronto, ON, Canada; 4 Department of Psychiatry, University of Toronto, Toronto, ON, Canada; 5 Department of Laboratory Medicine and Pathology, Temerty Faculty of Medicine, University of Toronto, Toronto, ON, Canada; 6 Leslie Dan Faculty of Pharmacy, University of Toronto, Toronto, ON, Canada; 7 Department of Medicine, University of Toronto, Toronto, ON, Canada; 8 Department of Critical Care, Faculty of Medicine, Dalhousie University, Halifax, NS, Canada; 9 Faculty of Medicine, University of Ottawa, Ottawa, ON, Canada

**Keywords:** artificial intelligence, medical education, health professions education, professionalism, ethics, equity, consensus methods, Delphi technique, responsible artificial intelligence, responsible AI

## Abstract

**Background:**

Artificial intelligence (AI) is rapidly integrating into health professions education and clinical practice, creating significant opportunities alongside new ethical challenges. Although current international and professional guidance establishes essential values, it offers limited direction for how clinicians, educators, learners, and institutions should act in routine educational, research, and clinical contexts. The CARE-AI (Contextual, Accountable, Responsible, and Equitable Artificial Intelligence) project responds to this practice-level gap by articulating guidance that moves beyond values toward professional accountability and equity, with explicit attention to educational, research, and clinical practice contexts.

**Objective:**

The study objective was to develop and validate a consensus-based, actionable framework of principles to guide responsible AI use across health professions education, research, and clinical care.

**Methods:**

We conducted a 3-phase modified Delphi consensus study, reported in accordance with the Accurate Consensus Reporting Document. Phase 1 involved 2 international professional meetings and 3 purposively sampled focus groups (AI or technology, health professions education, and ethics or professionalism) to adapt and refine draft principles using an exploratory qualitative approach. Phase 2 used an online survey with a 5-point importance scale and prespecified consensus criteria (inclusion ≥70%: high ratings; exclusion ≥70%: low ratings). Phase 3 used include, exclude, or undecided voting on revised principles. Quantitative thresholds determined consensus. Qualitative free-text comments informed iterative refinement.

**Results:**

Participants represented diverse communities of practice across health professions education, clinical care, patient partners, ethics, and digital health, spanning multiple professional roles and training levels. Across all phases, 303 unique participants contributed to the study. Phase 1 focus groups (n=61) provided early insight and direction. In phase 2, the first Delphi survey round, 242 participants initiated the survey, with 120 (49.6%) participants completing it. In phase 3, the second Delphi survey round, 103 participants were invited based on expressed interest at the end of the first round; 78 participants initiated the survey and 75 completed it (75/78, 96.2% of starters). In phase 2, of the 61 statements, 58 (95%) met the inclusion criteria, and participants submitted 1887 comments (697 were content rich), prompting clearer accountability language, stronger equity commitments, and more usable wording. In phase 3, all 10 principles and their statements met the inclusion criteria. Participants contributed 224 comments (179 were content rich) that informed final refinements. Endorsement was near unanimous: 96% (72/75) agreed or strongly agreed that the framework clearly defined professionalism expectations for AI to meet educational, technological, and ethical needs in the health professions.

**Conclusions:**

The Health CARE-AI Framework, with its preamble and 10 principles, articulates actionable, consensus-validated guidance that moves from values to competence, into professional accountability, and toward structural commitments to equity. Paired with a companion implementation guide and toolkit, the framework is intended to support use across education, research, and clinical settings.

## Introduction

The integration of artificial intelligence (AI) into health care systems has ushered in a new era of medical practice and administration, a transformative development that is reshaping the landscape of health care. These technologies hold great promise for enhancing patient care, improving outcomes, and streamlining processes. However, the rapid adoption of these technologies also presents a unique set of challenges, often unintended, particularly in the realm of professional ethics and conduct. To navigate this transformation responsibly, health care professionals need clear guidance and practical frameworks for using AI in ways that safeguard patient welfare, uphold professional values, and nurture public trust [1-4].

Several international organizations have issued AI ethics frameworks that provide important high-level guidance. The United Nations Educational, Scientific, and Cultural Organization’s (UNESCO) “Recommendation on the Ethics of AI” emphasizes human rights, fairness, transparency, and human oversight across sectors, including education, science, culture, public administration, and health [5]. The Organisation for Economic Co-operation and Development (OECD) AI Principles highlight human-centered values, transparency, robustness, and accountability, and have been adopted across government, industry, and public-sector governance frameworks since 2019 [6]. The World Health Organization (WHO) has outlined broad ethical principles for AI in health, including human autonomy, transparency, equity, and accountability [1], and its more recent regulatory considerations reinforce these priorities for national systems [7]. These frameworks provide important foundations but are not designed for use in busy daily clinical workflows or the specific needs of health professions education (HPE), including supervision, assessment, and the relational dimensions of care and learning, defined here as trust-based interactions among patients, learners, educators, and care teams [1,5-7].

Professional bodies and academic consortia have begun to interpret these principles for medicine and medical education. The American Medical Association’s policy on augmented intelligence outlines expectations for safety, bias mitigation, transparency, and preservation of clinical autonomy [8]. The Association of American Medical Colleges (AAMC) has issued principles for the responsible use of AI in and for medical education, emphasizing transparency, equitable access, interdisciplinary collaboration, and human-centered focus in teaching and learning [9]. It has also released separate guidance for the use of AI in medical school and residency selection, focusing on issues of bias, validity, and equitable access [10]. While these initiatives highlight the urgency of addressing AI in education, they remain early consensus statements rather than frameworks that have been systematically validated in practice. A recent AAMC curriculum snapshot across Canada and the United States documents rapid AI uptake alongside variability and a lack of common standards [11].

Reviews and guidance documents on AI implementation in medical education consistently point to fragmented efforts and limited evidence. Peer-reviewed articles on generative AI highlight both opportunities for innovation and risks to integrity and professionalism, yet empirical evidence to guide implementation remains scarce [4]. Scoping reviews describe heterogeneous curricula with wide variation in how AI is introduced, a lack of common standards, and little evaluation of long-term outcomes [2]. Systematic reviews reinforce these concerns, identifying persistent gaps in validated competencies and measurable outcomes [12].

Together, these observations indicate that available frameworks explain why ethics matter and what values ought to guide AI, but they do not resolve how clinicians, educators, and learners should act in specific contexts or embed accountability, equity, and relational trust in day-to-day decision-making. This gap created the rationale for our study—Contextual, Accountable, Responsible, and Equitable Artificial Intelligence (CARE-AI)—which sought to develop principles tailored to HPE, research, and clinical care through a structured international consensus process.

## Methods

### Design and Purpose

**Figure 1.**
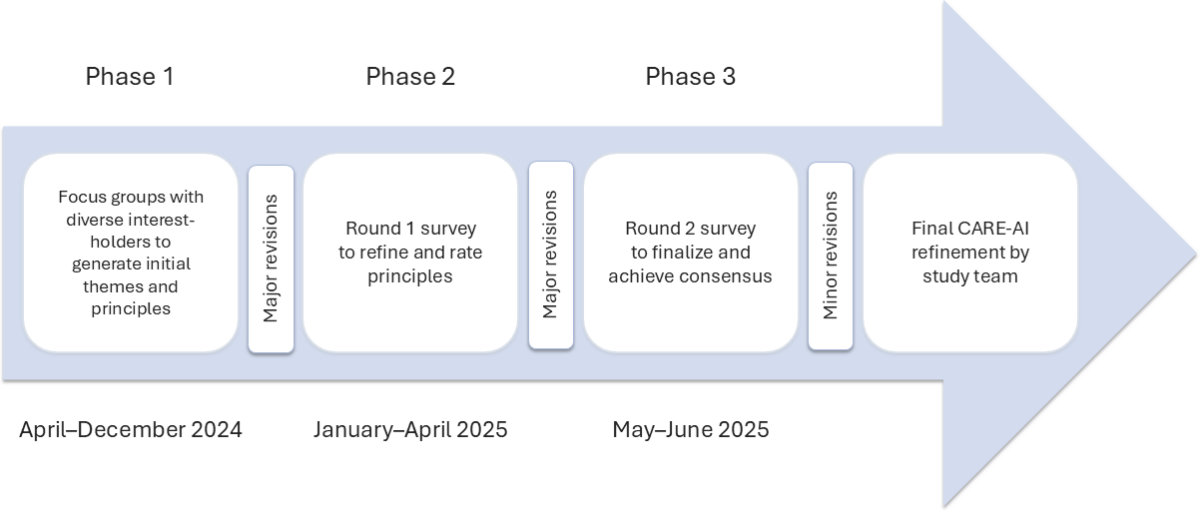
Project phases and timeline. CARE-AI: Contextual, Accountable, Responsible, and Equitable Artificial Intelligence.

The Equity in Health Systems (EqHS) leadership team (LKS, JM, DW, VD, BT, BJ, MM, and BH) conducted a 3-phase, modified Delphi study to develop and validate principles for accountable and responsive AI ethics in health care and across the health professions learning continuum (Figure 1). A modified Delphi was selected to support structured, iterative consensus where definitive empirical standards are limited and expert judgment is warranted [13,14]. Reporting follows the Accurate Consensus Reporting Document guideline for consensus methods [15].

### Participants and Recruitment

Purposive sampling sought breadth across roles and perspectives. To ensure epistemic diversity, recruitment was organized around three predefined domains of expertise: (1) AI and technology, (2) HPE, and (3) ethics and professionalism. Participants included clinicians, educators, and learners, as well as patient partners and caregivers, who contributed lived-expertise perspectives alongside their additional expertise in AI, technology, or ethics; members of professionalism offices and institutional bodies, who had experience overseeing professional conduct and ethics; and health equity experts, who specialized in health care optimization and addressing systemic disparities in health systems. Eligibility required current or recent engagement with AI in health or education and willingness to participate in one or more phases. Invitations were sent via email by the research team, initially distributed through EqHS, the AAMC Group on Information Resources, the International Association for Health Professions Education Technology Enhanced Learning community, the Temerty Centre for Artificial Intelligence Research and Education in Medicine, and the Canadian Association for Medical Education technology working group, with snowballing encouraged [16].

### Phase 1: Building the Foundation

The first phase began from a position of listening. Rather than drafting principles in isolation, the team drew on established social media professionalism guidelines and invited critique and reimagining in the context of AI [17]. Two professional meetings became early testing grounds for this work: the International Summit on Leadership Education for Physicians (2024; theme: *ethical integration of AI in health education*) and the Academy for Professionalism in Health Care (2024; theme: *enhancing professionalism through effective communication: from in-person to AI*).

Phase 1 used an exploratory qualitative approach to inform early principle development. Semistructured group discussions and focus groups with leaders across AI and technology, HPE, and ethics and professionalism were used to surface shared concerns, practical challenges, and gaps in existing guidance related to AI use. Discussions were oriented toward identifying areas of convergence across roles and contexts, with particular attention to where existing digital professionalism and social media guidelines failed to address AI-specific ethical issues. Analytic emphasis was placed on pragmatic, inductive pattern recognition and sense-making sufficient to identify areas of convergence, practical concerns, and implications for subsequent principle development and consensus testing rather than on formal thematic analysis [18].

Building on these insights, 3 focus groups, aligned with the domains of AI and technology, HPE, and ethics and professionalism, were convened. Using semistructured prompts, facilitators encouraged participants to identify priorities and surface risks and describe practical contexts in which the principles would need to be applied. Sessions were recorded and supported by detailed notes.

The study team reviewed recordings and notes using a pragmatic, inductive approach consistent with a constructivist descriptive framework, emphasizing close engagement with participant perspectives and the integration of lived and professional expertise. Analysis focused on identifying recurring concepts, areas of convergence, and practical challenges across groups. These insights were synthesized through iterative team discussion, where overlapping ideas were consolidated and translated into draft principle statements. The principles were refined for clarity and applicability across contexts, resulting in an initial set of draft principles that were carried forward into the modified Delphi process.

### Instrument Development and Piloting

An online survey was built in Research Electronic Data Capture (REDCap; Vanderbilt University), an electronic data capture platform widely used in health research (Multimedia Appendix 1) [19,20]. Items asked participants to rate each draft principle on appropriateness and actionability and to provide free-text comments on clarity, scope, feasibility, and equity or relational considerations. The instrument was refined through cognitive interviewing with coauthors and a small group of collaborators to optimize wording, anchors, and flow prior to fielding [21].

### Phase 2: Round 1 Survey

Round 1 opened with a brief preamble describing the aims and intended use of the framework, followed by item-level ratings and comment boxes for each principle. Participants rated importance on a 5-point Likert scale anchored from unimportant to extremely important and could identify gaps, justify ratings, and suggest wording refinements. A priori consensus was defined as follows: include if at least 70% of respondents rated 4 to 5 and fewer than 15% rated 1 to 2, exclude if at least 70% rated 1 to 2, and no consensus flagged for modification and rerating [13,14]. Free-text comments were reviewed descriptively and mapped to each principle. Even when inclusion thresholds were met, qualitative input guided wording changes to strengthen clarity and accountability.

### Phase 3: Round 2 Survey

Round 2 presented the revised preamble and principles for focused judgment with 3 response options: include, exclude, or undecided. Items with at least 70% “include” were retained and items with at least 70% “exclude” were removed. Panelists received an anonymized summary of group feedback and revisions between rounds. Where participant feedback improved clarity or feasibility without altering intent, wording was adjusted.

### Data Integration and Rigor

While quantitative thresholds determined provisional inclusion or exclusion, qualitative commentary was systematically integrated to sharpen scope, specify accountability, and strengthen equity and relational dimensions. Rigor was supported by *a priori* definitions of objectives, panel composition and recruitment strategy, scoring approach and consensus rules, number of rounds, and stopping criteria; controlled feedback between rounds; investigator review of borderline items; and documentation of wording changes across iterations, consistent with Accurate Consensus Reporting Document recommendations for transparent reporting of consensus methods [15].

### Ethical Considerations

This study was reviewed and approved by the University of Alberta Research Ethics Board, Edmonton, Canada (level 2, study ID Pro00143413). Participation was voluntary and anonymous through survey rounds. Participants did not receive compensation for their involvement in the study.

## Results

### Phase 1: Building the Foundation

The CARE-AI principles were informed by social media professionalism guidelines [17]. Initial feedback from the two professional meetings made it clear that AI was not a continuation of social media best practices. These sessions engaged 24 participants, whose input guided the need for ethical, privacy, and bias considerations to be central to any AI framework. Three subsequent focus groups (n=37: AI, n=12; HPE, n=14; and ethics, n=11) broadened this base of understanding. Participants included clinicians, educators, administrators, ethicists, AI specialists, and patient and caregiver partners predominantly from Canada and the United States. Collectively, they generated 10 draft principle statements and provided extensive feedback on the framing preamble.

Feedback from these meetings underscored both the urgency of addressing AI’s ethical challenges and the need for a framework that could speak across professions, training levels, and contexts. The early preamble emphasized individual judgment and institutional values, positioning CARE-AI as “professionalism principles with a focus on ethical, privacy, and bias challenges posed by AI technologies.” By the end of phase 1, participants had urged a more expansive framing, one that clarified what “AI” means, recognized diverse roles, and emphasized collective responsibility. These suggestions set the stage for considerable refinement in later phases.

### Phase 2: Delphi Round 1

A total of 242 participants initiated the round 1 survey, with 120 completing it (completion rate: 49.6%). Participants represented a wide spectrum of roles, including patient partners (Table 1). Additionally, participants were able to select multiple roles. Geographically, most were from Canada (68/120, 56.7%) and the United States (39/120, 32.5%), with smaller representation from Europe, Asia, Australia, the Caribbean, and Africa.

Of the 61 statements rated, 58 (95%) surpassed the prespecified consensus threshold and none required removal. Participants contributed 1887 comments, 697 of which were content-rich. These qualitative data prompted significant revisions: clarifying accountability language, embedding equity more explicitly, and adjusting tone to ensure accessibility. Accountability-related comments also highlighted expectations for responsible data use and stewardship. Each line item was modified based on the feedback provided. By the close of round 1, 89% (110/124) of respondents agreed or strongly agreed that the draft principles effectively outlined professionalism expectations for AI.

**Table 1.** Distribution of participant roles among respondents in Delphi rounds 1 and 2^a^.

Role	Phase 2 participants (round 1), n (%)	Phase 3 participants (round 2), n (%)
AI^b^ specialist	104 (43.7)	41 (54.7)
Clinician	107 (45)	41 (54.7)
Education leader	117 (49.2)	44 (58.7)
Education technology specialist	96 (40.3)	32 (42.7)
Health equity expert	58 (24.4)	24 (32)
Health professions educator	153 (64.3)	53 (70.7)
Patient or caregiver partner	49 (20.6)	19 (25.3)
Professionalism office	50 (21)	16 (21.3)
Researcher	121 (50.8)	45 (60)

a Roles were multiselect, so percentages exceed 100%.

b AI: artificial intelligence.

### Phase 3: Delphi Round 2

In round 2, of the 103 participants invited, 78 initiated the survey, and 75 completed it (completion rate: 75/103, 72.8% of those invited, 75/78, 96.2% of starters). The panel reflected a similar distribution to phase 2. Participants were primarily from Canada (47/75, 62.7%) and the United States (22/75, 29.3%), with additional representation from the United Kingdom, Europe, Asia, Australia, and the Caribbean. All principles presented in round 2 achieved inclusion consensus (≥70% agreement). Participants submitted 224 comments, of which 179 were substantive, guiding final refinements. The preamble was expanded to include what we mean by AI, the need for iterative self-reflection, and the interconnectedness of AI.

### Evolution of a Principle

The progression of principle 1 illustrates how participant input shaped the framework (Table 2). In the earliest draft developed after the conferences, the principle was expressed as “AI is a personal responsibility,*”* highlighting judicious use and the need for critical human review of AI outputs. By the end of phase 1, following focus group feedback, this was reframed as a matter of “professional duty,” urging individuals to handle information carefully, recognize how AI could perpetuate bias, and act as ethical stewards.

**Table 2.** Progression of principle 1 across study phases.

Study phase	Principle 1
Phase 1 (after conferences)	Curation of information is a personal responsibility. Handle information with care, especially when using AI^a^ to train, generate, or process data. Understand that AI systems can unintentionally perpetuate and amplify existing biases in data. Apply this knowledge to guide your use of AI, ensuring that it promotes fairness and equity in health care settings.
Phase 1 (after focus groups)	Responsible AI is a professional duty. As a professional, it is your individual responsibility to maintain knowledge and ethical use of AI systems. Your role may span institutions and contexts; you are expected to align with their organizational policies related to AI. You remain an advocate for evaluating, selecting, and implementing appropriate AI tools. You are also responsible for refining their performance to align with the values of health care and accountability to patients, team members, and health care systems.
Phase 2 (after Delphi round 1)	Responsible AI use is both an individual and collective duty. Individuals, teams, and institutions contribute to the safe and equitable use of AI in health settings by establishing expectations, developing supportive policies, and regularly reviewing tools to ensure alignment with health care values. As professional contributions often span institutions and contexts, expectations and standards may vary. Stay informed about guidelines, apply best practices in AI use, and help refine policies as new use cases and concerns emerge. AI integration must remain grounded in a shared commitment to safe, responsible use, centered on patients^b^, learners, care partners, and the broader health community.
Phase 2 (after Delphi round 2)	Responsible AI use is both an individual and collective duty. Individuals, teams or groups, and institutions contribute to the safe and equitable use of AI in education, research, and health care settings by establishing expectations, developing supportive policies, and regularly reviewing resources to ensure alignment with health care values. Stay informed about guidelines as they may vary between institutions and contexts. AI integration must be grounded in a shared commitment to safe and responsible use, centered on patients^b^, learners, and the broader health community.

a AI: artificial intelligence.

b We use the term “patients” inclusively to encompass individuals receiving care as well as their caregivers and others directly involved in supporting their health and well-being.

In phase 2, participants stressed that responsibility extended beyond individual behavior to include institutional alignment, policy awareness, and accountability to patients, teams, and systems, shifting the wording to underscore shared responsibility. The final version emphasized that individuals, teams, and institutions collectively contribute to the safe and equitable use of AI in education, research, and health care, and that accountability must remain grounded in a shared commitment to patients, learners, and the broader health community.

This trajectory demonstrates how most substantive shifts occurred between phases 1 and 2, when participants pressed for clearer professional and institutional accountability. By phase 3, refinements were more incremental, polishing language for clarity and inclusiveness.

### Final Framework

**Figure 2.**
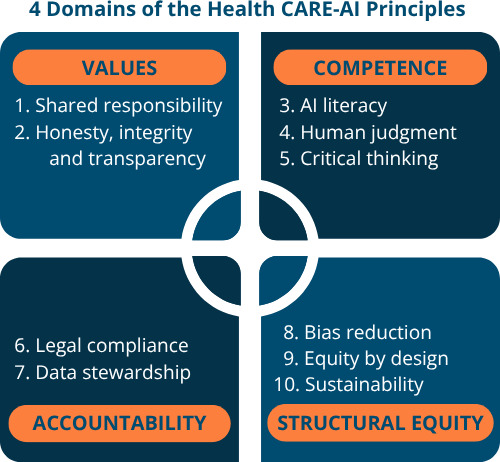
Conceptual model of the Health Contextual, Accountable, and Responsible Ethics for Artificial Intelligence (CARE-AI) principles. AI: artificial intelligence.

Endorsement for the final framework was near unanimous: 96% (72/75) of participants agreed or strongly agreed that it clearly articulated the professionalism expectations required for AI to effectively address the educational, technological, and ethical needs within the health professions (agree: 43/75, 57.3%; strongly agree: 29/75, 38.7%). The Health CARE-AI Framework, shown in Figure 2, brings together a preamble and 10 guiding principles that move deliberately from foundational values to structural equity. Across all phases, participant feedback sharpened the language of both the preamble and the principles, reinforcing clarity, strengthening accountability, and embedding equity. The framework is organized into 4 interdependent domains (values, competence, accountability, and structural equity) that chart a trajectory from individual behaviors to systemic change. Figure 2 presents the Health CARE-AI principles as 4 interrelated domains rather than a sequential process; visual proximity reflects conceptual relationships rather than hierarchy or order of implementation. The full framework is found in Multimedia Appendix 1, with the high-level summary presented in Textbox 1.

Textbox 1. **A high-level summary of the Health Contextual, Accountable, and Responsible Ethics for Artificial Intelligence Framework principles.**

1. Responsible artificial intelligence (AI) use is both an individual and collective duty. This clarifies responsibilities at individual, team, and institutional levels; sets expectations for reviewing tools; and keeps patients and learners at the center.
2. Use AI with honesty and integrity. Be transparent about AI assistance, avoid deception or manipulation, and disclose use in learning and care in line with policy.
3. Build and maintain AI literacy. Commit to ongoing, role-appropriate learning, resource training accordingly, and feed experience back to improve local policies and practice.
4. Responsible AI use should complement, not replace, human judgment. Retain professional accountability and verify AI outputs against clinical and educational judgment.
5. Think critically before speaking, acting, or uploading with an AI system present. Treat AI as a present third party, anticipate persistence and misinterpretation, and choose words and uploads that protect relationships and trust.
6. Work within the law. Follow privacy, consent, and intellectual property requirements, and do not delegate legally reserved tasks to AI.
7. Use and share information ethically in AI-supported environments. Understand how data are collected, stored, and shared; obtain appropriate consent; and safeguard privacy and data sovereignty.
8. Use AI in ways that actively reduce bias and promote equity. Check outputs for bias, report concerns, support auditing and updates, and adapt use as models and data change.
9. Build equity into AI foundations. Embed equity in design and governance, ensure accessibility, use diverse data, and co-design with affected communities throughout development and evaluation.
10. Advance sustainable AI in health systems. Use AI in ways that are proportionate to benefit; mindful of impacts on patient care, the workforce, and the environment; and that strengthen rather than erode people and systems.

## Discussion

### Principal Findings

This study used a multiphase consensus process to develop and validate the Health CARE-AI Framework to bridge the gap between traditional health care ethics and the unique challenges posed by AI. Across conferences, focus groups, and modified Delphi rounds, participants refined a preamble and 10 principles that move from foundational values to structural commitments to equity. All 10 principles reached inclusion in round 2, with strong endorsement (72/75, 96% agreed or strongly agreed). Qualitative comments sharpened accountability language, strengthened equity, and improved clarity for education and clinical use.

The Health CARE-AI Framework is designed for integration into education and governance. Within curricula, educators can map existing professionalism and digital health teaching against the 10 principles. At the institutional level, the principles can guide policy language, supervision protocols, and oversight of AI initiatives. Barriers to implementation exist and include uneven faculty readiness, high workload, and policy lag. An integration toolbox is likely needed for success.

The framework aligns with major reports while addressing practice-level gaps. Relative to BEME Guide No. 84, which maps the evolving integration of AI in medical education, our work moves beyond descriptive synthesis toward actionable expectations for educators and learners across classroom and clinical contexts [2]. It similarly operationalizes AAMC principles by translating high-level commitments such as transparency, equity, and human-centered use into role-specific behaviors that can be explicitly taught, supervised, and assessed within training and practice environments [9]. In parallel, the framework renders foundational values articulated by UNESCO, OECD, and WHO, including human rights, accountability, robustness, and fairness, into concrete responsibilities that identify who is accountable, in which contexts, and under what conditions of oversight [5-7]. In doing so, CARE-AI bridges the gap between normative guidance and day-to-day professional practice, offering a consensus-based framework that supports implementation across diverse roles, settings, and stages of the health professions continuum.

Participants consistently emphasized 3 challenges: accountability, data stewardship, and equity. Principle 1 establishes responsibility at both individual and collective levels, while privacy and stewardship expectations are reinforced through commitments to ethical data use, transparency, and alignment with law and policy (principles 2, 6, and 7). Principles 8 and 9 address equity in complementary ways: users are called to mitigate bias in practice, and institutions are charged with embedding equity into system design. Some questioned whether principle 9 was necessary alongside principle 8, but consensus affirmed its importance for structural change rather than reliance on vigilant end users.

Participants raised environmental concerns, primarily related to the energy use and carbon footprint of large-scale AI models and infrastructure. This resulted in the development of principle 10 on sustainability, inclusive of workforce sustainability. Additionally, stewardship is embedded in shared responsibility (principle 1), lawful and ethical governance (principles 6 and 7), and equity by design (principle 9), with institutions considering sustainability in procurement and oversight.

Participants also emphasized how the principles required greater pregrounding. In response, we expanded the preamble to clarify scope, define AI, and underscore collective accountability. The preamble now explicitly addresses the environments in which the principles will be applied; recognizes the diverse interactions of patients, care partners, clinicians, educators, learners, researchers, developers, and administrators; and addresses the need for inclusive dialogue, transparency, collaboration, and co-design in the implementation process.

### Implications for Implementation and Practice

We offer 2 practical starting points for implementation. First, embedding the principles into existing professionalism expectations in HPE ensures learners encounter them early and repeatedly. Second, incorporating them into program and institutional governance provides a common standard for committees overseeing AI initiatives such as clinical decision support, admissions processes, and educational technologies.

To help educators and organizations embed these principles into contexts, we have created a practical implementation guide and toolkit, which includes 4 illustrative scenarios for use in teaching and governance, and targeted faculty development sessions [22]. This planning resource supports leaders in health professions to proactively assess their current state, engage their teams, and implement values-aligned AI adoption strategies. Without such support, integration risks remaining aspirational. Validation of this implementation guide and toolkit is completed within the contexts of HPE, research, and clinical care. Engagement with professional organizations (eg, medical education, regulatory, and accreditation bodies) will be important to support broader dissemination, alignment with standards, and update of the framework. Future work should explore formal endorsement and collaboration with these organizations as a pathway to implementation at scale.

### Reflexivity and Limitations

While substantial qualitative feedback was collected across phases (eg, free-text survey comments and focus group discussions), we did not undertake a formal thematic analysis. Instead, qualitative data were synthesized using a pragmatic, inductive approach to inform iterative refinement of principles, with emphasis on clarity, accountability, and equity. As a result, the study does not provide a detailed qualitative account of participant perspectives or divergent viewpoints. This may limit insight into how different interest-holder groups interpreted or prioritized key issues and should be considered when interpreting the findings.

Most participants were from English-speaking, high-resource settings. Regions outside North America and contributors from remote locations were underrepresented. The study team was largely academic, though it included learners, clinical leaders, frontline clinicians, and researchers, all from different career stages, which strengthened reflexivity and interpretive breadth. Additionally, participants represented diverse roles (eg, clinicians, educators, AI specialists, ethicists, and patient partners), which strengthen the study but may also have introduced role-related biases. However, it is worth noting that representation of patient and caregiver partners had lower representation and therefore may influence the perspectives reflected in the final framework. Those in ethics or equity may have emphasized risks and safeguards, while those in AI-focused roles may have prioritized feasibility and implementation. Although the Delphi process aimed to balance these perspectives, role-based priorities may have influenced the framing and weighting of principles, resulting in a negotiated consensus across groups rather than uniform perspectives.

As with all consensus-based approaches, panel composition shapes the perspectives represented, and iterative rating processes may attenuate minority or dissenting views. Apparent agreement may therefore reflect convergence on least-objectionable wording rather than uniform strength of endorsement across participant groups. Findings should be interpreted as consensus among those who participated at this time and revisited as technologies, professional norms, and implementation contexts evolve.

Methodological and practical constraints also warrant consideration. Selection and attrition bias are possible, as approximately half of the participants who initiated round 1 completed the survey; reasons for noncompletion were not formally assessed. To preserve clarity and durability of the framework, suggestions to incorporate extensive cross-references or illustrative examples were intentionally deferred, with applied scenarios provided separately in the implementation toolkit. Institutional and policy constraints may also limit frontline agency, reinforcing participant emphasis on the need for clearly articulated responsibilities and accountability across individual, organizational, and system levels during implementation.

### Conclusions

This study developed and validated the Health CARE-AI Framework through a multiphase consensus process shaped by diverse communities of practice. The final framework, comprising a preamble and 10 principles, received strong endorsement and reflects careful refinement across phases. Taken together, the principles emphasize equity and professional accountability as core considerations for AI use across the health professions, progressing from values to competence and toward structural commitments to equity.

The framework addresses professionalism expectations for AI use across the health professions in practical ways. Educators can anchor curricula and faculty development; learners can guide day-to-day decisions in study and supervised practice; and programs and health systems can align policies, governance, and quality improvement to safeguard patient welfare, uphold professional integrity, and nurture public trust.

Building on this work, we have developed a companion implementation guide and toolkit with scenario-based applications across education, research, and clinical contexts, which has undergone high-level validation prior to rollout. We invite programs to integrate the framework, evaluate its use, and share outcomes. Future work should examine uptake, fidelity, and impacts on equity and trust, and update the framework as technologies and contexts evolve.

## Supplementary material

10.2196/91626 Multimedia Appendix 1 Full framework of the Health CARE-AI (Contextual, Accountable, Responsible, and Equitable Artificial Intelligence) principles.

## Abbreviations

AAMC: Association of American Medical Colleges
AI: artificial intelligence
CARE-AI: Contextual, Accountable, and Responsible Ethics for Artificial Intelligence
EqHS: Equity in Health Systems
HPE: health professions education
OECD: Organisation for Economic Co-operation and Development
REDCap: Research Electronic Data Capture
UNESCO: United Nations Educational, Scientific, and Cultural Organization
WHO: World Health Organization
