# Supplementary material for: Principles for Responsible AI in Health Professions Education, Research, and Care: Health CARE-AI (Contextual, Accountable, Responsible, and Equitable Artificial Intelligence) Framework Delphi Consensus Study
Source: JMIR Med Educ. 2026 Jul 22;12:e91626. doi: 10.2196/91626 (PMC13395757; doi:10.2196/91626)

Santé Bruyère Institut de recherche | Bruyère Health Research Institute

Future Skills Centre | Centre des Compétences futures

EqHS Equity in Health Systems

HEALTH CARE-AI

A Framework for Contextual, Accountable, Responsible & Equitable Artificial Intelligence

in Health Professions Education, Research, and Care

Everyone in healthcare, across training stages, professions, and roles, will be impacted by artificial intelligence (AI). The HEALTH CARE-AI (Contextual, Accountable, Responsible, and Equitable Artificial Intelligence) Framework provides guiding principles for the ethical and safe use of AI in healthcare, health professions education, and research. Individuals must adopt ethical behaviors, apply critical thinking, and remain open to learning as these technologies evolve and augment human capabilities.

What We Mean by AI. In this framework, artificial intelligence refers broadly to computer systems that perform tasks traditionally requiring human intelligence, including reasoning, prediction, pattern recognition, and decision-making. While AI encompasses a range of technologies such as machine learning, expert systems, and rule-based algorithms, this framework focuses on generative, evaluative, and predictive systems. These include tools that create or analyze text, audio, video, images, or algorithmic outputs, such as large language models (LLMs), image generators, speech synthesizers, and clinical decision support systems. Although the capabilities and risks of AI vary across modalities, the ethical responsibilities outlined here apply in any context where AI influences, augments, or automates human activity.

Using AI responsibly requires **a continuous commitment to doing one's best** throughout an iterative process of learning, critical reflection, and advocacy. While decisions are shaped by personal judgment, they must be grounded in shared professional values. Responsible ethics goes beyond following rules. It involves actively considering the consequences of our choices, anticipating potential risks, and making informed, accountable, and transparent decisions. AI tools should protect dignity, support autonomy, and be applied in ways that promote equity and do more good than harm.

Ethical practice in AI-supported environments depends on recognizing the diverse roles, responsibilities, and perspectives of those involved. **AI is shaped by the interconnected actions** of patients, care partners, clinicians, educators, learners, researchers, developers, and administrators. Decision-making should be rooted in collective accountability and shaped through inclusive dialogue. Transparency, collaboration, and co-design across contexts help ensure that AI is developed and applied in ways that reflect shared values and serve the broader health community.

The CARE-AI principles are **intentionally organized to follow a progression from foundational values to structural impact.** They begin with shared responsibility and the importance of building AI literacy. From there, the focus shifts to individual use, professional judgment, and ethical behavior. Legal compliance and privacy come next, grounding ethical practice in clear obligations. The final principles emphasize bias mitigation and the need for equity in both the use and design of AI systems. **Together, these principles move from values to competence, through action and accountability, and toward long-term systemic change.**

Ethical AI use in healthcare depends on conscious choices,
collective responsibility, critical thinking, and a commitment to equity and integrity.

CARE-AI Principles

1. **Responsible AI use is both an individual and collective duty.** Individuals, teams or groups, and institutions contribute to the safe and equitable use of AI in education, research, and healthcare settings by establishing expectations, developing supportive policies, and regularly reviewing resources to ensure alignment with healthcare values. Stay informed about guidelines as they may vary between institutions and contexts. AI integration must be grounded in a shared commitment to safe and responsible use, centered on patients*, learners, and the broader health community.
2. **Use AI with honesty and integrity.** Maintain transparency and ethical conduct in all AI-related activities. Do not use AI for deceitful purposes, such as impersonation, manipulation, or the spread of misinformation. Disclose AI involvement in academic work and patient care decisions, in accordance with relevant policies.
3. **Build and maintain AI literacy.** Commit to ongoing professional development to understand AI's evolving capabilities and limitations as it pertains to your professional practice. Apply best practices in AI use and provide feedback as new use cases and concerns emerge to refine policies within the scope of your expertise. Fostering environments where AI knowledge and skills are resourced, utilized, and supported is a collective responsibility.
4. **Responsible AI use should complement, not replace, human judgment.** AI must not supersede professional responsibilities in decision-making related to teaching, learning, research, and patient care. Critically review AI-generated outputs before use, as they may be incomplete or incorrect, and do not overestimate their credibility.
5. **Think critically before speaking, acting, or uploading with an AI system present.** What is said, done, or entered may become part of the enduring AI landscape. AI may not accurately differentiate nuances such as diction, humour, or sarcasm, and may misinterpret ambiguous or informal language as facts. Be aware of the impact AI tools have on patient interactions. AI tools function as present third parties, potentially influencing what patients and healthcare team members say when they know their words are being transcribed.
6. **Work within the law.** Follow laws and policies related to AI use, including those governing intellectual property, copyright, data privacy, and institutional requirements. While laws guide practice, ethics must lead in areas where legal frameworks lag. Individuals are responsible for knowing where to access this information and whom to consult for clarification. Institutions are responsible for providing this information. Understand when consent or permission is required before inputting data or documents into AI systems, and act accordingly. Do not delegate tasks to AI that are legally required to be completed by a human. Be aware of the potential legal and ethical consequences of how information is stored and circulated through AI platforms. When using AI tools on personal devices in professional contexts, ensure that their use complies with institutional and legal standards.
7. **Use and share information ethically in AI-supported environments.** Ensure a clear understanding of how AI systems collect, use, and store data. Protect the rights of individuals, including learners, research participants, and patients, by obtaining appropriate consent, safeguarding privacy, and respecting data sovereignty. Do not release content that is not intended to be public facing.
8. **Use AI in ways that actively reduce bias and promote equity.** AI systems can unintentionally perpetuate inequity and amplify existing biases. To mitigate these risks, evaluate AI outputs for bias and guide their refinement when possible. Remain aware of recent AI tool updates, including when systems were last refreshed or trained on data, as outdated inputs can reinforce historical bias. Report concerns about biased outputs through appropriate channels. Support regular audits and updates and provide ongoing feedback to strengthen AI performance over time.
9. **Build equity into AI foundations.** Develop AI systems in ways that embed equity at every stage of design and implementation. Embedding equity includes addressing systemic barriers, ensuring accessibility and inclusivity for all users, and involving those most impacted in design, testing, and evaluation. Proactively use diverse and reliable data to build AI systems and engage affected communities in participatory approaches and decision-making. Equity must guide both the process and the outcome.
10. **Advance Sustainable AI in Health Systems.** Sustainable AI use requires understanding and assessing AI's affordability and long-term impacts on patient care, the workforce, and the environment. Decisions to use AI should be proportionate to the intended benefit and potential harm. AI should not be used to normalize unsafe workloads, inappropriately substitute human care, or obscure systemic resource limitations. AI should strengthen the systems and people it is intended to support.

** We use the term 'patient(s)' inclusively to encompass individuals receiving care as well as their caregivers and others directly involved in supporting their health & wellbeing.*

Four Domains of the Health CARE-AI Principles

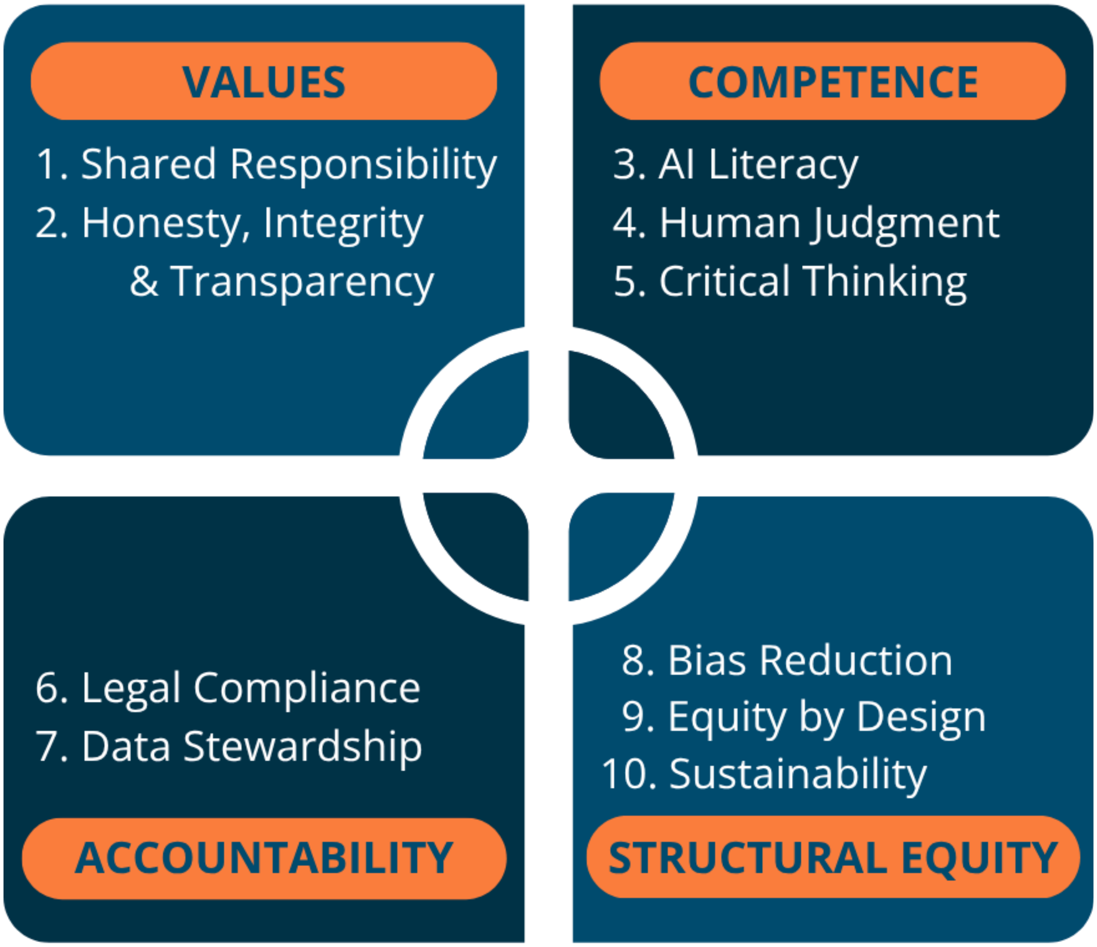

Supplement: Multimedia Appendix 1 [file mededu-v12-e91626-s001.pdf]
